# Treatment of osteoporosis with denosumab in patients with decreased kidney function

**DOI:** 10.1007/s11657-023-01306-x

**Published:** 2023-07-26

**Authors:** Ole Lander Svendsen

**Affiliations:** 1 Department of Endocrinology, Bispebjerg and Frederiksberg Hospital, Copenhagen University Hospital, Ebba Lunds Vej 44, Entrance 60, ground floor, DK-2400 Copenhagen, NV Denmark; 2 Department of Clinical Medicine, Faculty of Health and Medical Sciences, University of Copenhagen, Copenhagen, Denmark

**Keywords:** Osteoporosis, Fractures, Chronic kidney disease, Decreased kidney function, Denosumab

## Abstract

**Introduction:**

Little is known about treatment of osteoporosis with denosumab (Prolia®) in patients with decreased kidney function. The aim of this retrospective case report study was to investigate effects and side-effects of such treatment.

**Methods:**

Since 2012, 75 patients with osteoporosis and decreased kidney function had been treated with denosumab (Prolia®) in the osteoporosis outpatient clinic of the department of endocrinology, Bispebjerg Hospital, University of Copenhagen, Denmark, and data were retrospectively collected from the patient records of these patients in 2021.

**Results:**

At baseline, the mean estimated glomerular filtration rate (eGFR) was 34 mL/min (range 9–50) and the median age was 85 years (range 45–103). 95% of the patients had had low-energy fractures, and the bone mineral density *T* score of the hips was on average − 2.7. All, but one, patients had normal/high parathyroid hormone (PTH) levels.

The mean duration of the treatment with denosumab at the follow-up was 5.3 years (range 1.5–10). There was an annual increase of 12% and of 7% in the *T* score of in the lumbar spine and hip, respectively, compared to the *T*-scores prior to the denosumab treatment. 20% had a new fracture during the follow-up. 21% had biochemical hypocalcemia following denosumab injection, 7% developed symptoms of hypocalcemia, whereas 4% needed to be hospitalized acutely.

**Conclusion:**

Treatment with denosumab of osteoporosis in patients with decreased kidney function (eGFR 9–50 mL/min), with normal/high PTH, seems in general to be well tolerated, with improvement of bone and decreased risk of new fractures.

## Introduction

Patients with decreased kidney function and chronic kidney disease (CKD) have an increased risk of osteoporosis and fragility fractures [1, 2]. Drugs used for treatment of osteoporosis have been investigated in, and are primarily approved for, patients with normal kidney function. Measurements of bone mineral density (BMD) and biochemical markers of bone turnover have not been considered valid in patients with CKD. Furthermore, the risk of promoting adynamic bone disease with depressed bone turnover by anti-resorptive osteoporosis drugs, as well as extra skeletal deposition of calcium in blood vessels, i.e., vascular calcifications, has been of concern [3]. However, recent consensus emphasizes the need for treatment of osteoporosis in patients with CKD to decrease their high risk of fractures [4].

Denosumab is approved for treatment of osteoporosis, as Prolia® 60 mg injected subcutaneously every 6 months [5]. Denosumab is not eliminated renally and can as such be used in patients with decreased kidney function. But there are limited studies of denosumab in patient with decreased kidney function with an estimated glomerular filtration rate (eGFR) < 30/mL/min [5]. Safety issues of special concern are risk of hypocalcemia immediately after the injection of denosumab [6], as well as increased risk of fractures of vertebral bodies after discontinuation of denosumab [7].

Since 2012, patients with osteoporosis, that had decreased kidney function, were offered treatment with denosumab (Prolia®^)^) in the osteoporosis outpatient clinic of the department of endocrinology, Bispebjerg Hospital, University of Copenhagen, Denmark, and the aim of this study was to follow-up on the effect and side-effects of the treatment with denosumab of these patients. Calcium and vitamin D3 supplementation were prescribed unless contraindicated.

## Patients

All patients at the out-patient osteoporosis clinic of the department of endocrinology at Bispebjerg Frederiksberg hospital, Copenhagen University Hospital, Denmark, who had been treated with denosumab (Prolia®^)^) as first line therapy from 2012 and until 2021, were identified through the patient administrative systems. Inclusion criteria for the present study was treatment of osteoporosis with denosumab due to decreased kidney function. The exclusion criteria included lack of documentation in the hospital records, as well as if treatment with denosumab was selected due to either side effects, contraindications, or treatment failure of bisphosphonates or teriparatide, and lastly, if denosumab had been chosen as treatment without known reason.

## Methods

An observational study with retrospective data collection from patient records in 2021 of the patients included.

Bone mineral density (BMD) *T* scores of the anteroposterior lumbar spine and the hips had been assessed with a Lunar Prodigy DXA-scanner (GE, Madison, WI, USA, with encore 2005 software, version 9.15.010).

Serum levels of ionized-calcium (Ca), phosphate (P), parathyroid hormone (PTH), and kidney function by eGFR (creatinine was measured and the estimated glomerular filtration rate was calculated routinely by the lab) at baseline and once up to 14 days following the injection of denosumab were registered from the patient records. In case of hypocalcimia, additional follow-up measurements performed.

## Results

145 patients were identified. 71 patients were excluded for the following reasons: 16 were excluded due to missing data, 3 had never received denosumab, 48 were excluded because they were treated with denosumab due to side-effects (*n* = 19), treatment failure (*n* = 15), or contraindication (*n* = 14) to bisphosphonates, 2 were treated due to side-effects (*n* = 1) or treatment failure (*n* = 1) of teriparatide, and no reason given (*n* = 1).

Thus, 75 patients were identified having received denosumab treatment at least once in the outpatient osteoporosis clinic due to decreased kidney function and were included in the study.

### Baseline

The mean eGFR was 34 mL/min (range 9–50 mL/min). As can be seen in the Table 1, 61% of the patients had an eGFR of 30–44 mL/min and 25% had an eGFR of 15–29 mL/min. 3 patients were in hemodialysis, and one had a transplanted kidney.

**Table 1:** Distribution of patients in different categories of eGFR and CKD stage [8]

eGFR (mL/min)	45–59^1^	30–44	15–29	< 15^2^
CKD stage (8)	G3a	G3b	G4	G5
Number of patients	9	46	19	1

^1^Highest eGFR was 50 mL/min and ^2^lowest eGFR was 9 mL/min

At first visit, the median age of the patients was 85 years (range 45–103). The female/male ratio was nearly 2:1. The mean age was 84 years and 81% were females in those with eGFR < 30 mL/min compared to 85 years and 47% females in those with eGFR > 30 mL/min.

Prior to treatment with denosumab, the mean BMD T-scores were –2.0 in the lumbar spine, and –2.7 of the hips. 95% of the patients had had low-energy fractures, and some had had multiple fractures. 79% of the fractures were of vertebral bodies or of the hips.

36% of the patients had arteriosclerotic disease (ischemic heart disease, myocardial infarction, stroke, and peripheral arterial deficiency) prior to the treatment with denosumab.

### Follow-up

As shown in Table 2, all but one had PTH within or above the upper limit of the reference range of the assay. 15% had a high phosphate, whereas 44% had serum calcium levels below the lower limit of the reference range.

**Table 2:** Number of patients with low, normal, high, or missing values compared to the reference limits of the assays

	Parathyroid hormone (PTH)	Phosphate	Calcium-ion
Low	1 (1%)	2 (3%)	33 (44%)
Normal	21 (28)	54 (72%)	35 (47%)
High	47 (63%)	11 (15%)	6 (8%)
Missing	6 (8%)	8 (11%)	1 (1%)

PTH: 1.1–7.1 pmol/L; phosphate: 0.71–1.23 mmol/L; calcium-ion: 1.18–1.32 mmol/L

The mean duration of the treatment with denosumab at the follow-up was 5.3 years (range 1.5–10 years). 62 (83%) patients remained on denosumab, whereas 13 (17%) patients had discontinued the treatment. Of those who discontinued, four were due to decrease in kidney function, five due to hypocalcemia, two due to non-attendance, and one chose to discontinue due to age (99 years old). None received other treatments for osteoporosis after discontinuation of denosumab.

DXA scans were available for 27 patients, 6 men, and 21 women, at follow-up. 12 patients had falsely elevated BMD *T*-scores due to degenerative changes in the lumber spine and, therefore, were excluded from the analysis. Thus, the mean BMD *T*-score of the lumbar spine was − 2.6 and of the hip − 2.7 at the follow-up, which corresponds to an annual increase of 12% and of 7% in the BMD *T*-score in the lumbar spine and the hip, respectively, compared to the BMD *T*-scores prior to the denosumab treatment.

15 patients (20%) had new fractures during the follow-up. Of these, twelve patients were still treated with denosumab. Three patients had new fractures after discontinuation of denosumab. One patient had a low-energy fracture of T12 a few months after discontinuation of denosumab, another patient had a low-energy Colle’s fracture 2 years, and a knee and shoulder fracture 4 years, after discontinuation of denosumab, whereas the last patient had a low energy-fracture of the pelvis, ribs, and the clavicular 4 months after the discontinuation of denosumab.

### Hypocalcemia following the denosumab injection

16 (21%) patients had biochemical hypocalcemia following the denosumab injection. Of these, most patients were asymptomatic (calcium ion 0.99 to 1.16 mmol/L). 5 (7%) patients developed symptoms of hypocalcemia (calcium ion 0.82 to 1.03 mmol/L), such as peripheral paraesthesia and vomiting, and 3 (4%) of these patients needed to be hospitalized acutely. The calcium ion in the hospitalized patients was 0.82 to 0.99 mmol/L, and the eGFR was 28, 38, and 45 mL/min, respectively. 5 patients had discontinued the treatment with denosumab due to hypocalcemia, three due to hospitalization, one due to symptoms, and one due to asymptomatic hypocalcemia (calcium ion 0.99 mmol/L).

### Atherosclerotic disease

One patient had had vascular surgery due to peripheral arterial deficiency a month after first administration of denosumab, whereas another patient was diagnosed with angina after 2½ years of treatment with denosumab. Three patients had strokes, after 3 months, 1½ year, and 7 years of treatment with denosumab, respectively, and one patient had an acute myocardial infarction after 2 years of treatment with denosumab. Four out of these six patients had atherosclerotic disease prior to the treatment with denosumab.

### Mortality

23 patients (31%) had passed away at the follow-up in 2021. These patients had been treated with denosumab for mean 1.7 years. Cause of death of special interest was complications after low-energy hip fractures (*n* = 2), cardiac arrest (*n* = 2), acute myocardial infarction (*n* = 2), stroke (*n* = 1), and heart failure (*n* = 1). Most of the cardiovascular deaths occurred in patients with known cardiovascular disease (5 out of 6).

## Discussion

The present observational retrospective study with a long follow-up period is the largest case report study so far of treatment of osteoporosis with denosumab in patients with decreased kidney function and normal/high serum parathyroid hormone level, i.e., without adynamic bone. If a patient in our osteoporosis clinic had biochemical sign of adynamic bone, the patient was conferred with a dedicated nephrologist, and if suspicion of adynamic bone, treatment with denosumab, or other specific treatment of osteoporosis, was not initiated.

Given the expected very high risk of fractures in this population, only 20% of the patients had a new fracture during the follow-up.

Furthermore, in those patients that had a DXA scan during the follow-up, the BMD *T*-scores did improve with an annual increase of 12% in the lumbar spine and 7% in the hip.

Regarding safety, 21% had biochemical hypocalcemia immediately after injection of denosumab, of which 7% had symptoms, and 4% of the patients were hospitalized. The incidence of cardiovascular disease and mortality with denosumab treatment was not higher than expected in this high-risk population [9].

Only one patient had a fracture of a vertebral body despite the risk of bone loss [7] after discontinuation of denosumab.

Limitations of this study is lack of a control group, missing data due to the retrospective review of the patient records, and the fact that 61% of the patients had an eGFR ≥ 30 mL/min (30–50 mL/min). However, the results seem somewhat in line with previous results in patients in hemodialysis [10, 11].

## Conclusion

Treatment of osteoporosis with denosumab in patients with decreased kidney function (eGFR 9–50 mL/min), with normal/high PTH levels, seems in general to be well tolerated, with improvement of bone and decreased risk of new fractures.
